# Large metastatic lymph nodes misdiagnosed as a pancreatic tumor: a case report

**DOI:** 10.1093/jscr/rjaf583

**Published:** 2025-08-01

**Authors:** Qiong Duan, Yanan Huang, He Li

**Affiliations:** Department of General Surgery, Affiliated Zhongshan Hospital of Dalian University, No. 6 Jiefang Road, Zhongshan District, Dalian City 116001, PR China; Department of General Surgery, Affiliated Zhongshan Hospital of Dalian University, No. 6 Jiefang Road, Zhongshan District, Dalian City 116001, PR China; Department of General Surgery, Affiliated Zhongshan Hospital of Dalian University, No. 6 Jiefang Road, Zhongshan District, Dalian City 116001, PR China

**Keywords:** early gastric cancer, lymph nodes, metastasis, case report

## Abstract

Due to the anatomical proximity between pancreatic tumors and the group 8 lymph nodes of the stomach, misdiagnosis can easily occur. The patient’s preoperative examination: computed tomography (CT) scan examination indicated an abdominal mass, 3.0T pancreatic magnetic resonance imaging (MRI) with contrast enhancement showed a pancreatic head lesion, possibly a neuroendocrine tumor. Gastroscopy revealed highly suspicious for cancer. Pancreaticoduodenectomy was planned. However, intraoperative exploration revealed the tumor to be localized within the Group 8 lymph nodes rather than the pancreas. Radical distal gastrectomy, along with resection of the lymph node mass, was performed. Histopathological analysis confirmed early gastric cancer with metastatic lymph node involvement. This case highlights a diagnostic pitfall wherein gastric cancer with lymph node metastasis was mistaken for a pancreatic tumor due to their anatomical overlap.

## Introduction

Early gastric cancer is defined as cancer involving the mucosa or submucosa, regardless of lymph node metastasis. The 5-year survival rate of early gastric cancer patients after radical surgery is over 90% [1, 2]. Many related studies in the literature have shown that multiple factors affect recurrence and survival, with lymph node metastasis being one of the most closely associated factors. The survival rate of patients with or without lymph node metastasis differs by approximately 8% [3]. The highest incidence of lymph node metastasis in early gastric cancer occurs in lymph node station No. 3, followed by No. 4 and No. 6, while No. 8a has a lower incidence of metastasis, these lymph nodes are located at the head of the pancreas, are closely related, and can be easily confused with pancreatic tumors [4].

## Case report

**Figure 1:**
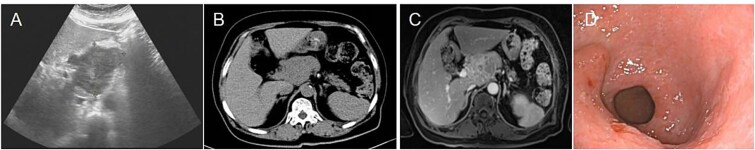
Preoperative imaging and endoscopic findings. (A) Abdominal ultrasound identified a space-occupying lesion located in the upper abdominal region. (B) Abdominal CT scan reveals a mass located in the head of the pancreas. (C) 3.0T MRI with contrast enhancement revealed a mass located in the pancreatic head, indicating a potential malignant lesion. (D) Electronic gastroscopy showed chronic atrophic gastritis and antrum bulge.

A 74-year-old female patient with a 15-year history of diabetes presented with uninduced foot numbness that began 6 months ago and worsened 1 week prior to admission. Upon hospitalization, a mass was identified in the upper abdomen. Laboratory tests revealed a blood glucose level of 6.80 mmol/l, neuron-specific enolase (NSE) at 18.34 ng/ml, carcinoembryonic antigen (CEA) at 9.69 ng/ml, and a white blood cell count of 10.0 × 10^9^/l. An abdominal computed tomography (CT) showed a mass in the pancreatic head, suspected to be a neuroendocrine tumor, with malignancy not excluded, along with retroperitoneal lymph node enlargement and atrophy of the pancreatic body and tail; the patient had a history of gallbladder surgery. Pancreatic enhanced magnetic resonance imaging (MRI) (3.0T) confirmed an occupying lesion in the pancreatic head, suggesting a neuroendocrine tumor, with malignancy to be ruled out. Electronic gastroscopy indicated chronic atrophic gastritis and revealed a 2.5 × 2.0 cm flat hyperplastic lesion on the anterior wall of the gastric antrum and along the greater curvature (Fig. 1).

**Figure 2:**
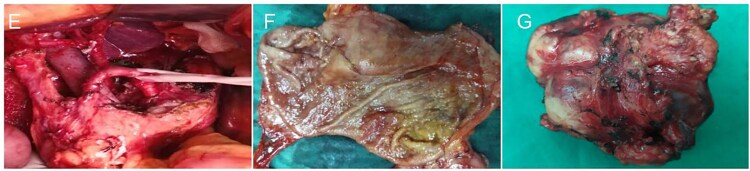
Intraoperative and gross pathological findings. (E) Intraoperative anatomical assessment revealed that the group 8 lymph nodes were densely adherent to the pancreatic parenchyma and adjacent vascular structures. (F) Gastric cancer tissue. (G) The surgically resected mass is located in the group 8 lymph node compartment.

**Figure 3:**
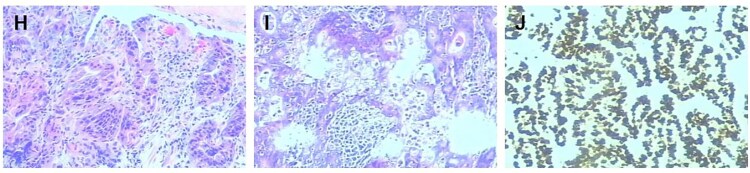
Histopathological and immunohistochemical analysis (×200 magnification). (H) The preoperative gastroscopic biopsy immunohistochemistry (×200) results were highly suspicious for malignancy. (I) Immunohistochemical analysis of the resected gastric carcinoma specimen (×200) supports the diagnosis of early gastric cancer. (J) Histopathological evaluation of the resected specimen confirmed metastatic involvement of the lymph node tissue (×200).

The diagnosis of gastric cancer was confirmed, and the possibility of a malignant pancreatic tumor could not be excluded. Surgery was performed on 20 June 2021, with the preoperative plan involving pancreaticoduodenectomy and partial gastrectomy. Intraoperative findings revealed no abdominal ascites and no abnormal nodules in the peritoneum, liver, colon, or small intestine, and no tumor invasion in the stomach. A 6 × 5 cm mass was identified in the lymph node station of group 8, characterized by a hard texture, limited mobility, and close association with surrounding blood vessels, while no obvious thickening was seen in the antrum (Fig. 2). Scattered lymph nodes were found around the stomach. The tumor was located at the upper origin of the pancreas and could be separated from the pancreatic tissue. Based on intraoperative findings, early gastric cancer with lymph node metastasis was suspected. Consequently, radical distal gastrectomy and tumor resection were performed. Postoperative pathology showed that group 8 lymph nodes measured 7.5 × 5.3 × 4.0 cm and appeared gray and brittle. The partial gastrectomy specimen measured 13.0 × 4.5 × 3.5 cm. Gastric intramucosal carcinoma (well-differentiated, Fig. 3H) was identified, with no evidence of vascular tumor emboli or nerve invasion. Surgical margins and the omentum were free of cancer. Two metastatic lymph nodes were found in Group 8, while no metastasis was detected in the remaining lymph nodes. Immunohistochemical analysis of the gastric mass showed C-erbB2 (2+), Ki-67 index 90%, CK7 (focal +), CK20 (−), CA125 (−), CA199 (−), Villin (+), and CDX2 (+). For group 8 lymph nodes, results were CK7 (+), CK20 (−), CA125 (−), CA199 (−), Villin (+), and CDX2 (+) (Fig. 3I and J).

## Discussion

Early gastric cancer was first proposed by the Japanese Gastric Cancer Association and is defined as a tumor limited to the mucosa and submucosa, regardless of lymph node metastasis [5]. More than 80% of early gastric cancer patients do not have lymph node metastasis, and second-tier lymph node involvement is even rarer [6–9]. In this case, the diagnosis of early stage gastric cancer has been clearly established. A large mass was identified at the superior margin of the pancreas, despite discussions between radiologists and clinicians, the origin of the tumor remained unclear. Correlating preoperative gastroduodenoscopy findings with elevated tumor markers (CEA: 9.69 ng/ml, NSE: 18.34 ng/ml), it was considered that this case represented synchronous primary cancers, involving both a pancreatic neuroendocrine tumor and early gastric cancer. However, pancreatic tumor markers (CA19-) were within normal limits, while the Group 8 lymph nodes demonstrate close anatomical proximity to the pancreatic head. Some experts considered that the large tumor was suspected to be a lymph node metastasis from early gastric cancer. Intraoperative exploration ultimately revealed that the tumor was located in the Group 8 lymph nodes, and postoperative pathology confirmed that the large tumor near the upper margin of the pancreas was a metastatic lymph node originating from early gastric cancer. Due to the close anatomical relationship and proximity between the lymph nodes of the Group 8 lymph nodes and the head of the pancreas, it is challenging to determine the primary tumor site between the two based on imaging alone. In this report, the huge lymph node metastasis in Group 8 was not clearly differentiated from pancreatic tumors, atypical metastatic sites can lead to diagnostic confusion and misguide clinicians. In pancreatic space-occupying lesions, gastroscopy and endoscopic ultrasound are essential to exclude gastric primaries. Contrast-enhanced CT/MRI may misidentify Group 8 lymphadenopathy as direct pancreatic tumor invasion. A comprehensive approach (endoscopic, radiographic, and serologic evaluation) combined with multidisciplinary team collaboration enhances diagnostic accuracy and therapeutic decision-making.

## Data Availability

All data was available.
